# Reducing unwarranted variation in low-value health care in Norway: description of the NOR programme

**DOI:** 10.1007/s43999-026-00089-1

**Published:** 2026-05-28

**Authors:** Ole Tjomsland, Christina Drewes, Halvor Langeland, Kim Andre Loftheim Noremark, Christian Thoresen, Berte Bøe, Andreas Kristensen, Kristin Matre Aasarød, Line Strømhaug Grongstad, Elisabeth Pedersen, Marit Herder, Eva Stensland

**Affiliations:** 1 South Eastern Norway Regional Health Authority, Hamar, Norway; 2 University of Stavanger, Stavanger, Norway; 3 Northern Norway Regional Health Authority, Bodø, Norway; 4 Central Norway Regional Health Authority, Stjørdal, Norway; 5 Western Norway Regional Health Authority, Stavanger, Norway; 6 Oslo University Hospital, Oslo, Norway; 7 University Hospital of North Norway, Tromsø, Norway; 8 St. Olavs hospital, Trondheim University Hospital, Trondheim, Norway; 9 Centre for Clinical Documentation and Evaluation (SKDE), Tromsø, Norway; 10 UiT The Arctic University of Norway, Tromsø, Norway

**Keywords:** Low-value health care, Unwarranted clinical variation, Health care system intervention, National quality improvement programme, Clinical utilisation patterns

## Abstract

Multiple advice-based interventions designed to reduce the use of low-value procedures, i.e. procedures that are medically unnecessary because they provide minimal or no net health benefit, have so far demonstrated limited impact on utilisation rates. The underlying assumption for such initiatives is that increased knowledge, together with data demonstrating unwarranted variation, are sufficient to change clinical practice. Notably, this has not been the case. Numerous studies have documented complex organisational, professional, and patient-level barriers to change. Therefore, we advocate for sharing the experiences from initiatives designed to reduce low-value health care. The Norwegian Operational Group for Reassessment (NOR) Programme is a Norwegian national initiative, launched by the Chief Executive Officers of the Norwegian Regional Health Authorities and governed by the Centre for Clinical Evaluation and Documentation, to reduce the use of low-value procedures. Selection criteria included: (i) international designation as low-value care (e.g., Choosing Wisely, Evidence-Based Intervention Programme), (ii) moderate to high patient risk, (iii) high cost, and (iv) high volume and geographical variation. Based on these criteria, three initial targets were identified: (a) arthroscopic treatment of acromial impingement and repair of non-traumatic rotator cuff rupture; (b) upper gastrointestinal endoscopy in patients aged < 45 years; and (c) invasive coronary angiography in patients with stable coronary artery disease. Expert groups for each area were established in 2024 and have produced consensus-based recommendations describing a set of multifaceted interventions to reduce overuse. This manuscript presents those recommendations and shares early experience from implementation within the NOR Programme.

## Introduction

Low-value health care (LVC) is defined as medical services which, according to the best available evidence, deliver marginal or no net health benefit relative to their costs and risks [1]. Environmental costs have also been proposed as part of this assessment [2]. Reducing wasteful, unnecessary, or harmful care is therefore integral to improvement efforts that benefit patients and society [3].

Over recent decades, advice-based interventions, such as Choosing Wisely [4] and the Evidence-Based Intervention (EBI) Programme [5] have aimed to lower the overuse of LVC. While these have raised awareness and placed LVC firmly on the agenda, their measurable impact on utilisation rates has been limited [6–8].

Many LVC-targeted interventions rest on the assumption that knowledge dissemination, combined with data demonstrating overuse and unwarranted variation, will prompt change in clinical practice. Notably, this is rarely the case. Numerous studies describe obstacles to disinvestment and change across public and private health care systems [9, 10]. Consequently, we believe it is imperative that interventions designed to reduce LVC have an organisational multicomponent design to be effective.

Unwarranted variation, within and between regions, may both indicate overuse of unnecessary services as well as underuse of necessary ones [1] and can serve as a practical screening tool to detect lack of consensus or non-adherence to best practice.

### Aim

The aim of this paper is to describe the Norwegian Operational Group for Reassessment (NOR) Programme’s method for selecting low-value procedures amenable to national, multifaceted interventions to reduce utilisation rates, and to present the initial recommendations and share learning points from planning the implementation.

## The NOR programme

In 2023, the Chief Executive Officers (CEOs) of Norway’s four Regional Health Authorities (RHAs) launched a national programme, governed by the Centre for Clinical Documentation and Evaluation (SKDE), to reduce LVC. The Chief Medical Officers (CMOs) in the RHAs were appointed as the steering committee. The programme’s goal is to reduce unnecessary examinations and treatments within and between regions [11].

### Selection criteria

The NOR Group applied the following criteria to identify initial targets:

1. Procedures or services labelled as LVC by international expert groups (e.g., Choosing Wisely, EBI Programme).
2. Moderate to high patient risk.
3. High cost.
4. High volume and geographical variation.

Potential procedures were selected from the top 30 procedures ranked by cost and volume, as displayed in the clinical dashboard and identified using the criteria described above. Unwarranted variation was assessed using SKDE’s national health atlases and the executive dashboard, with a focus on regions where more than 50% of observed variation could not be explained by case mix. From this long list, three procedures were prioritised by the Steering Committee following a multi-criteria deliberation process.

### Tools for identification

The South-Eastern Norway Regional Health Authority (HSØ) has developed a dashboard featuring a limited number of quality indicators intended for executive boards and top-level leaders to monitor unwarranted variation in quality, patient safety and utilisation rates. The dashboard displays medical utilisation rates across all publicly reimbursed hospitals in Norway [12]. Together with national health atlases developed by SKDE [13], this dashboard was used to identify services with high unwarranted regional variation.

### Measurement and evaluation

Regional variation was assessed using indirectly standardised rates (age and sex) mainly derived from the Norwegian Patient Registry and supplemented by data from the Norwegian Registry of Invasive Cardiology (NORIC) in the coronary angiography (CAG)-project. Planned evaluations include utilisation trends, equity impacts (by sex, age, region), and balancing measures such as shifts to non-surgical care. Unintended consequences will be monitored via patient-reported measures where available.

### Multidisciplinary task forces

Each expert group included clinicians with relevant clinical specialties, general practitioners, methodologists and analysts. The work was presented and discussed with patient representatives from all four RHAs. Private for-profit and non-profit hospitals participating in publicly funded care were invited where relevant to ensure system-wide applicability of recommendations.

### Selected initiatives

Applying the established criteria, the NOR group selected three procedures for focused intervention:

- Arthroscopic acromial resection for impingement and repair of non-traumatic rotator cuff rupture.
- Upper gastrointestinal (GI) endoscopy in patients aged < 45 years.
- Coronary angiography in patients with stable coronary artery disease.

The regional CEOs approved further investigation, aiming to develop consensus on a national action plan to reduce overuse. Multidisciplinary task forces (expert groups) with representatives from all four RHAs were established for each initiative. Their initial goal was to produce consensus-based recommendations to reduce utilisation rates; these recommendations will be evaluated by the steering committee of NOR consisting of the CMOs in the RHAs and finally by the CEOs in the RHAs before they are implemented through national action plans.

## Overview of the Norwegian health care system and its impact on surgical volumes

Norway’s publicly funded universal health care system serves approximately 5.5 million inhabitants. The country is divided into four health regions, each overseen by an RHA responsible for delivering specialist health care through health trusts, including private for-profit and non-profit hospitals, which are independent legal entities with their own boards and management, responsible for the services they provide.

Most medical and surgical consultations and procedures, whether performed in public or private hospitals, or by contract specialists, are reimbursed by the RHAs, providing oversight and influence over care provision.

Although Norway faces comparatively fewer capacity constraints than some other systems, de-implementation is still warranted to reduce harm, release capacity for higher-value care, and contribute to environmental sustainability. Challenges identified internationally (e.g., limited impact of awareness campaigns alone) motivated the multi-component design of the NOR Programme. Decisions regarding the implementation and de-implementation of treatments deemed high- or low-value are made by the National Decision Forum (Beslutningsforum) together with the National Collaborative for New Methods (Sekretariatet for Nye Metoder). Decisions are based on illness severity, treatment utility, and cost-effectiveness [14]. The NOR Programme operates within this framework: NOR develops consensus-based recommendations and implementation plans which are submitted to the RHAs’ CMOs (steering committee) and CEOs for decision. NOR has no decision-making authority itself; rather, it provides the analytic basis, proposed targets and tools for change, and coordinates cross-regional execution once decisions are taken.

### Expert group recommendations

Expert groups for each initiative were appointed in 2024. Each expert group was asked to draft a consensus-based report containing recommended utilisation rates, and an action plan to reach the desired level, within their field of expertise. The reports were presented to the steering committee (the CMOs in the RHAs) to ensure that implementation of national action plans would both have bottom-up and top-down support.

### Shoulder arthroscopy

In 2021, National Decision Forum decided that acromial resection should not be offered as standard treatment for impingement syndrome. In 2023, the Forum advised that conservative treatment should be the first-line option in patients with degenerative rotator cuff rupture, sufficiently tested before any surgical intervention [15].

Despite these guidance updates, which were expected to reduce shoulder arthroscopy volumes, utilisation rates remained relatively stable. Norwegian Patient Registry data show a 23% decrease in acromial resection utilisation (from 69 to 53 per 100,000 inhabitants) over 2019–2022; nonetheless, 3,340 procedures were still performed in 2023. In the same year, ~ 4,400 cuff-repair procedures were conducted; the utilisation rate decreased by only 4% (from 77 to 74 per 100,000) over the last decade, despite the publication of randomised trials [15]. Substantial variation within and between regions was observed. Although national volumes of acromial resections declined after 2020, volumes of other arthroscopic shoulder procedures remained relatively stable. It is debated whether “creative coding” or increased use of other surgeries—some with limited cost-effectiveness evidence—partly explains this trend.

#### Key findings and recommendations shoulder arthroscopy

A multidisciplinary group—orthopaedic surgeons from all RHAs, a specialist in physical medicine, and a physiotherapist—met seven times (two in-person; five virtual). The group reviewed national utilisation, prior health technology assessments, and international guidelines. Discussions addressed referral practices, coding systems, surgical indications, and strategies to implement evidence-based care. Practical tools, including a treatment flowchart and a standardised rejection letter, were developed.

- Marked regional variation in surgical rates not explained by demographics or disease prevalence.
- Persistent use of procedures with limited or no documented benefit, despite national recommendations.
- Outdated national guidelines and training requirements for orthopaedic residents which may contribute to overuse.
- Effective interventions include:
  - Centralised referral systems.
  - Mandatory, adequately dosed physiotherapy before surgical consideration.
  - Termination of RHA-contracts with private commercial hospitals for shoulder surgery where appropriate.
- Coding practice variation affects monitoring and may incentivise combined procedures; coding revisions are recommended.
- Further centralisation of shoulder surgery to specialised centres may ensure adequate competence in both surgical and non-operative care.
- Establishment of a national audit committee is proposed to enhance consistency and accountability.
- Activity targets. No absolute national target was set; the current national average is recommended as an interim benchmark, with review after one year.

The report identifies substantial practice variation and systemic drivers of overuse in shoulder surgery. Recommendations emphasise strengthening conservative management, revising surgical indications, updating coding systems, and aligning the training requirements for orthopaedic residents with evidence-based practice. Implementation is expected to reduce unnecessary procedures, improve resource allocation, and enhance patient outcomes. Further evaluation and guidelines development are required to sustain long-term improvements [15].

### Upper GI endoscopy in patients aged < 45 years

Upper GI endoscopy was introduced in 1957 [16] for diagnosing and treating upper GI tract conditions and monitoring cancer-associated states in certain high-risk groups. Most conditions warranting gastroscopy are uncommon in persons aged < 55 years. In Norway, oesophageal and gastric cancers are very rare in this age group, and stomach cancer incidence has markedly decreased over the past seven decades [17].

The UK EBI Programme (Academy of Medical Royal Colleges) classifies upper GI endoscopy as an invasive procedure, often poorly tolerated, and carrying significant risks. EBI guidance recommends not using upper endoscopy as a first-line investigation in all patients, and restricts its use in those aged < 45 years to the following indications [18]:

- Any dysphagia (difficulty swallowing).
- Assessment of upper GI bleeding.
- Investigation of specific symptoms.
- Management of selected cases of *Helicobacter pylori*, Barrett’s oesophagus, and coeliac disease.

The “implementation categories” (patient pathway, governance tools, process management) structure the recommended actions and map to the topics addressed above (referral criteria, educational measures, feedback to referrers, and guideline alignment).

Upper GI endoscopy is invasive and may be poorly tolerated in some patients; recognised risks include bleeding, perforation, cardiopulmonary events related to sedation, post-procedural aspiration, and infection [18]. These risks are uncommon but increase with therapeutic interventions and co-morbidity. Including these risks aligns this section with the coronary angiography section’s framing of benefits and harms.

In Norway, upper GI endoscopy is among the most frequently performed procedures in publicly funded specialist care: ~112,000 procedures were conducted in 2022, with 27% in patients < 45 years and 41% in patients < 55 years. Significant regional variation, unexplained by disease prevalence or demographics, suggests potential overuse. Internationally, efforts are underway to reduce endoscopies in younger patients, whilst Norwegian guidelines currently set an age threshold of 45 years.

#### Key findings and recommendations upper GI endoscopy in patients aged < 45 Years

A gastroenterology expert group with representatives from all RHAs (six specialists in gastroenterology, one specialist in endocrinology and internal medicine, and one specialist in general practice) held nine meetings addressing:

- Age threshold. Maintain the 45-year threshold for now, aligned with national prioritisation guidelines and cancer care pathways.
- Referral practices. Current referral criteria rely heavily on outdated guidance issued by the Health Directorate and the Norwegian Electronic Medical Handbook (NEL); these should be revised and harmonised with international recommendations.
- Interventions. Use standardised rejection letters to referring general practitioners, provide feedback to high-referring clinicians, and implement educational measures as piloted at Sørlandet Hospital Trust [17].
- Clinical tools. Develop a flowchart for dyspepsia management and an information sheet for referrers; implement a test-and-treat strategy for *H. pylori* to reduce unnecessary gastroscopies.
- Functional dyspepsia. Improve terminology and public awareness; develop patient and clinician educational materials (currently lacking in Norway).
- Communication. Disseminate guidance via professional newsletters and podcasts to maximise engagement.

### Implementation categories

1. Patient pathway: Centralise referral units in each hospital area to align clinical practice; implement standardised information letters linked to clinical flowcharts containing educational resources on functional dyspepsia.
2. Update referral guidelines on NEL ( https://legehandboka.no/%3E) and Metodebok.no.
3. Governance tools: Reduce regional variation by aligning high-utilisation areas with national averages; evaluate after one year.
4. Process management: Ensure consistent interpretation of prioritisation guidelines across health trusts.

### Invasive examination and treatment of patients with chronic coronary artery disease

Traditionally, suspected chronic coronary disease has been assessed by exercise electrocardiogram and invasive CAG to confirm diagnosis, grade coronary pathology, and treat ischaemia-producing lesions with revascularisation (most commonly percutaneous coronary intervention, PCI). Recent evidence suggests that revascularisation in chronic coronary disease rarely improves prognosis over optimal medical therapy alone [19]. CAG is costly and carries procedural risks (e.g., bleeding, contrast-induced nephropathy, stroke, myocardial infarction, dissection, or death) [20]. Coronary Computed Tomography Angiography (CCTA), a non-invasive and generally well-tolerated modality, has emerged as the first-line investigation for individuals at low or intermediate risk of coronary artery disease [21]. According to NORIC approximately 1/3 of CAGs were performed to evaluate coronary artery disease in patients with “stable angina pectoris”. In these ~ 50% showed no significant epicardial stenosis.

The UK EBI Programme [21] and the 2024 European Society of Cardiology (ESC) Guidelines for Chronic Coronary Syndromes recommend CCTA as a first-line diagnostic tool for patients with low to moderate pre-test probability of coronary artery disease based on clinical examination, symptom characteristics and risk factors [22]. 

#### Key findings and recommendations: shoulder arthroscopy

The expert group consisting of five radiologists and five cardiologists from all RHAs held a total of seven meetings. Analysis of national data indicated that approximately 37% of all CAGs performed in 2022 yielded normal findings, with significant regional variation suggesting potential overuse. The report underscores the importance of collaboration between radiologists and cardiologists to ensure appropriate use of CCTA, particularly in chronic coronary syndromes. CCTA is recommended as the first-line diagnostic tool for patients with low to moderate pre-test probability of coronary disease, structural heart disease, and selected acute coronary syndrome cases. Standardised protocols and reporting using Coronary Artery Disease-Reporting and Data System (CAD-RADS) 2.0 [23] is recommended to improve diagnostic consistency and quality assurance.

Proposed measures include mandatory registration of CCTA examinations in NORIC, formal cooperation agreements between hospitals and private radiologic institutes, and development of national clinical guidelines. While no fixed national activity target is set, the group suggests that all institutions providing CAG should aim for a 5–10%-point reduction in diagnostic procedures not leading subsequent revascularisation.

The overarching aim is increased adherence to ESC guidelines [23] to reduce rates of invasive CAG in chronic coronary disease by increasing the use of optimal medical therapy and CCTA as the preferred initial investigation. Not all invasive procedures can be replaced by CCTA; diagnostic accuracy is limited in certain scenarios, and CCTA is a constrained resource (equipment and qualified interpreters). Changes in use of one diagnostic method should not lead to excess use of another.

### Interventions

- Standardised CCTA protocols. CCTA is performed in university-, local-, and private hospitals using different scanners. Standardisation, covering acquisition, reporting, and interpretation, is recommended, including use of CAD-RADS 2.0 [24]. Protocols should be adjusted to local practice/equipment to minimise referrals for further examinations. In patients without known coronary disease, pre-test likelihood should be calculated according to ESC 2024 guidelines, as part of pre-CCTA work-up.
- Comprehensive registration. All CCTA and CAG examinations should be registered in NORIC to enhance possibilities for quality improvement and research.
- Referral governance. Only cardiologists and specialists in internal medicine should refer for CCTA. Experienced cardiologists—preferably interventional—should allocate patients to invasive versus non-invasive pathways. Non-invasive centres should establish formal collaborations with interventional centres to ensure appropriate selection and follow-up.

## Discussion: preliminary learning points

Overarching lessons: (i) Governance – separating analytic recommendation from formal decision-making clarifies accountability and accelerates implementation; (ii) Process design – combining national dashboards, health atlases and specialty registries helps detect targets and monitor impact; (iii) Cross-cutting themes – standardised referral management, coding revisions, and clinician co-ownership recur across conditions and may generalise to other health systems with adaptations to local organisation and incentives.

### Barrier: fear of litigation

In addition to professional norms and patient expectations, fear of medico-legal consequences may incentivise defensive practice and contribute to persistent overuse. Regulatory clarity and supportive audit frameworks are required to mitigate this driver.

### Patient and general practitioner involvement

Despite decades of initiatives to reduce LVC, utilisation rates for many targeted interventions remain stable—in Norway as elsewhere. Roczniewska et al. [25] report that patient requests and physicians’ positive perceptions are strong predictors of LVC provision, with patient requests amplifying all other determinants. Therefore, interventions to reduce LVC need to address patient expectations to strengthen the gatekeeping role of general practitioners.

### Leadership involvement

Reducing unwarranted variation and LVC is a board-level responsibility. Yet executive boards and senior leaders face information overload from multiple sources, which can impede focus on specific areas of quality, safety, and utilisation rates. One remedy is periodic presentation of dashboards designed for executive boards and top-level leaders [12].

### Involving clinicians in setting recommended utilisation rates

The strategic aim to reduce unwarranted variation and LVC have not always been clearly communicated to leading clinicians. Thus, hospital staff may benefit from an information campaign that provides an understanding of drivers of variation together with practitioner involvement in finding an action plan that ensures compliance to best practice through responsibility for the problem and ownership for the solution [26].

Although population-level evidence may fail to show benefit, selected individuals may profit from treatment. National reduction aims may therefore be set stepwise, beginning with the lowest documented utilisation rates in regions without signs of underuse [14]. Also, regional utilisation rates from national health care atlases can be combined with a corresponding national medical quality registry that includes an assessment of clinical effect. This “dose-response relationship” of marginal effect can be explored to find optimal utilisation rates [27].

### Realistic estimation of savings: shift from low-value to high-value care

De-implementation is often assumed to reduce total volume, with savings equal to the volume and estimated average unit costs. However, ~ 84% of hospital expenditures are semi-variable or fixed [28]. Therefore, instead of cost savings, reducing low-value procedures may free capacity that can be redeployed to higher-value care. We emphasise shifting from low- to high-value care, rather than pursuing departmental budget cuts.

### National guidelines and reimbursement

Although Australia and the UK have adjusted recommendations to limit upper GI endoscopy in patients aged < 55 years, the Norwegian Health Directorate still recommends limited use in patients aged < 45 years [29], which differs from international thresholds. National recommendations should be aligned with international guidelines unless local data demonstrate otherwise; reimbursement policies should be updated accordingly.

In addition, regulatory authorities should contribute with an environment where fear of litigation and audits don’t drive the overuse of medical services.

### Using data across the clinical pathway

For a targeted casual intervention against LVC, Ganguli et al. [30] stresses the importance of identifying clinical pathways, i.e. cascades, that lead from consultation to unnecessary testing and referrals, that finally ends up as unwarranted treatment in the hospitals. Recently Tjomsland et al. [15] identified such a cascade within arthroscopy for shoulder pain. The study found minimal geographical variation in the number of patients consulting their general practitioner for shoulder complaints; however, there was considerable geographical variation in referral rates to specialist healthcare and even greater variation in the use of shoulder surgery. Moreover, arthroscopic shoulder procedures were performed more frequently in private than in public hospitals. Such pathway-level data are crucial when planning LVC reduction.

### Addressing the adverse effects of clinical subspecialisation

Increasing subspecialisation has affected medical staffing, particularly in rural hospitals. Surgical volumes have increased moderately, while the number of surgeons has grown substantially—due to working-time regulations and subspecialisation. Generalists capable of handling orthopaedic and general surgical emergencies have been replaced by specialists with narrower competence, potentially reducing flexibility to adjust surgical practice. In our work on reducing overuse of acromial resection and non-traumatic cuff repair, highest utilisation rates were observed in small rural hospitals with experienced shoulder surgeons but with limited general orthopaedic skills. We believe these surgeons should be offered re-training (e.g., in joint replacement) to avoid capacity-driven overuse. Currently, Norway has no recertification or Continuing Medical Education requirements to maintain the orthopaedic surgery specialty.

### Hospital size

One-third of the 49 Norwegian hospitals offering 24/7 emergency care in internal medicine and surgery can be characterised as small (catchment < 50,000 inhabitants). To sustain robust services, these hospitals require minimum staffing levels, particularly specialised doctors and nurses. Maintaining clinical and technical skills—and training residents and registrars—requires sufficient activity, especially in surgical specialties. Reducing LVC in such settings may create conflicts of interest, as down-scaling could threaten volumes needed to maintain and retain competence for acute care.

### Treatment versus lifestyle intervention

Obstacles to de-implementation vary by nature of the treatment. The introduction of PCI, a less invasive alternative to coronary artery bypass surgery (CABG), led to rapid uptake of PCI and reduced CABG volumes—representing a patient-preferred shift to less invasive care. By contrast, after national recommendations against standard use of acromial resection and repair of non-traumatic rotator cuff rupture, only marginal reductions in total shoulder arthroscopy were observed. Although the use of codes for acromial resection and cuff repair decreased, other procedures, not necessarily evidence-based, increased. We question whether patients with chronic musculoskeletal pain may prefer procedural over exercise-based and lifestyle interventions.

### Environmental implications

The anticipated reductions in procedures and patient travel suggest a favourable climate impact, which will be assessed upon completion of the implementation period.

### Limitations

The interventions in this protocol are implemented within Norway’s public health care system, where care is available to all regardless of income or geography. Approximately 20% of the population hold private health insurance, often employer-funded; interventions described here do not target privately insured hospital care. Our experience may not be directly applicable to countries with different health care organisation.

## Conclusion

The NOR programme is a Norwegian initiative aimed at reducing unwarranted variation and low-value health care. This manuscript describes the programme’s method for selecting low-value procedures and its strategy for implementing a national action plan. Three initial procedures—arthroscopic treatment of acromial impingement and repair of non-traumatic rotator cuff rupture; upper gastrointestinal endoscopy in patients aged < 45 years; and invasive coronary angiography in patients with stable coronary artery disease—have been evaluated, and consensus-based recommendations with multifaceted interventions have been developed. The effectiveness of these recommendations will be assessed.

## Data Availability

No data are available.

## Declarations

### Disclaimer

Data from the Norwegian Patient Registry have been used in this publication. The interpretation and reporting of these data are the sole responsibility of the authors; no endorsement by the Norwegian Patient Registry is intended or should be inferred.

### User involvement

The recommendations have been presented to the User Panels in all RHAs.

### Protocol

The manuscript describing the protocol is based on the revised Standards for Quality Improvement Reporting Excellence (SQUIRE 2.0).

## References

[1] Elshaug AG, Rosenthal MB, Lavis JN et al (2017) Levers for addressing medical underuse and overuse: achieving high-value health care Lancet 390(10090):191–202. 10.1016/S0140-67361632586-7. Epub 2017 Jan 9.PMID: 28077228 10.1016/S0140-6736(16)32586-7 28077228

[2] Barratt AL, Bell KJ, Charlesworth K et al (2022) High value health care is low carbon health care. F Med J Aust 216(2):67–68. 10.5694/mja2.51331. Epub 2021 Nov 14.PMID: 34699070 10.5694/mja2.51331 PMC9299213 34699070

[3] Sutherland K, Levesque JL (2020) J Unwarranted clinical variation in health care: Definitions and proposal of an analytic framework. Eval Clin Pract 26(3):687–696. 10.1111/jep.13181. Epub 2019 May 28 10.1111/jep.13181 PMC7317701 31136047

[4] Choosing Wisely https://www.choosingwisely.org / Accessed 13 Jan 2026

[5] Evidence-based interventions – academy of medical royal colleges (2026) (AOMRC) https://ebi.aomrc.org.uk/ 2026

[6] Cliff BQ, Acena A, Hirth RA et al (2021) Impact of choosing wisely interventions on low value medical services: a systematic review. Milbank Q 10.1111/1468-0009.12531 PMC8718584 34402553

[7] Farrar N, Conefrey C, Bell M et al (2025) Relevance and flexibility are key: exploring healthcare managers’ views and experiences of a de-adoption programme in the English National Health Service. BMC Health Serv Res 25590. 10.1186/s12913-025-12700-1 10.1186/s12913-025-12700-1 PMC12020301 40269905

[8] Anderson M, Molloy A, Maynou L et al (2022) Evaluation of the NHS England evidence-based interventions programme: a difference-in-difference analysis. BMJ Qual Saf 0:1–10. 10.1136/bmjqs-2021-014478 10.1136/bmjqs-2021-014478 PMC9887378 35393354

[9] Hofmann B (2021) Internal barriers to efficiency: why disinvestments are so difficult. Identifying and addressing internal barriers to disinvestment of health technologies. Health Econ Policy Law 16(4):473–488. 10.1017/S1744133121000037 33563362 10.1017/S1744133121000037

[10] Chambers JD, Salem MN, D’Cruz BN et al (2017) A review of empirical analyses of disinvestment initiatives. Value Health 20(7):1028–1036 Epub 2017 May 16 10.1016/j.jval.2017.03.015 28712620

[11] Langeland H, Tjomsland O, Panchakulasingam K et al (2025) E Tidsskr nor laegeforen. Unwarranted variation - warranted change 30;145(10). 10.4045/tidsskr.25.0308. Print 2025 Sep 9.PMID: 40923180 10.4045/tidsskr.25.0308 40923180

[12] Tjomsland O, Thoresen C, Ingebrigtsen T (2024) Reducing unwarranted variation: can a clinical dashboard be helpful for hospital executive boards and top-level leaders? BMJ Lead. 8(3):186–190. 10.1136/leader-2023-000749 10.1136/leader-2023-000749 PMC12038096 38053259

[13] Norwegian national health atlas https: https://www.skde.no/helseatlas/en/v2/kvalitet/. Accessed 15 Jan 2026

[14] Sekretariatet for Nye Metoder https://nyemetoder.no. Accessed 15 Jan 2026

[15] Tjomsland O, Bertilsson HM, Bjerkan G et al (2025) Protocol for a national intervention programme aimed to reduce unwarranted variation and overuse of shoulder arthroscopy in Norway. BMJ Open Qual 14(1):e003099. 10.1136/bmjoq-2024-003099 10.1136/bmjoq-2024-003099 PMC11843012 39979063

[16] Niwa H (2006) The History of Digestive Endoscopy. New Challenges in Gastrointestinal Endoscopy (s. 3–28), Springer Nature

[17] Hernes SS, Høiberg M, Gallefoss F et al (2024) Intervention for reducing the overuse of upper endoscopy in patients < 45 years: a protocol for a stepwise intervention programme. BMJ Open Qual 13(2):e002649. 10.1136/bmjoq-2023-002649. PMID: 38684346 10.1136/bmjoq-2023-002649 PMC11086486 38684346

[18] Academy of medical royal colleges (2022) Upper GI endoscopy - best practice guidance. Available: Upper GI endoscopy - EBI https://aomrc.org.uk

[19] Perera D, Clayton T, O’Kane PD et al (2022) Percutaneous revascularization for ischemic left ventricular dysfunction. N Engl J Med 387(15):1351–1360. 10.1056/NEJMoa2206606. Epub 2022 Aug 27 10.1056/NEJMoa2206606 36027563

[20] Gin J, Yeoh, Thijs V et al (2023) Coronary angiography complicated by acute ischaemic stroke and the use of thrombolysis: a cardiology perspective and narrative review of current literature. Curr Cardiol Reports 25:1499–1512 10.1007/s11886-023-01962-y 37847358

[21] UK EBI programme https://ebi.aomrc.org.uk/interventions/exercise-electrocardiogram-ecg-for-screening-for-coronary-heart-disease/10.1007/s12410-017-9412-6

[22] Moss AJ, Williams MC, Newby DA et al (2017) The updated NICE guidelines: cardiac CT as the first-line test for coronary artery. Dis Curr Cardiovasc Imaging Rep 10(5):15. 10.1007/s12410-017-9412-6. Epub 2017 Mar 27. PMCID: PMC5368205 10.1007/s12410-017-9412-6 PMC5368205 28446943

[23] ESC Scientific Document Group (2024) ESC Guidelines for the management of chronic coronary syndromes. Eur Heart J 45(36):3415–3537. 10.1093/eurheartj/ehae177 10.1093/eurheartj/ehae177 39210710

[24] Xu C, Sun Y, Wang L, Zou L et al (2025) Spectral Photon-Counting Detector Coronary CTA With Reduced Radiation and Contrast Medium Dose in Inflammation-Associated Coronary Artery Disease: A Prospective Study. Am J Roentgenol 225(6):e2533493 24 Dec 2025 40928168 10.2214/AJR.25.33493

[25] Roczniewska M, Augustsson H, Ingvarsson S et al (2025) Relative importance and interactions of factors influencing low-value care provision: a factorial survey experiment among Swedish primary care physicians. BMJ Qual Saf 2025. bmjqs-2024-018045 10.1136/bmjqs-2024-018045 PMC12418588 39947899

[26] Eide H, Barach P, Søreide E et al (2023) Managing unwarranted variation in hospital care – findings from a regional audit in Norway. Res Health Serv Reg 2:16. 10.1007/s43999-023-00033-7 39177902 10.1007/s43999-023-00033-7 PMC11281730

[27] Rudolfsen JH, Solberg TK, Ingebrigtsen T et al (2020) Associations between utilization rates and patients’ health: a study of spine surgery and patient-reported outcomes (EQ-5D and ODI). BMC Health Serv Res 20: 135 10.1186/s12913-020-4968-2 PMC7036171 32087710

[28] Roberts RR, Frutos PW, Ciavarella GG et al (1999) Distribution of variable vs fixed costs of hospital care. JAMA 281(7):644–649. 10.1001/jama.281.7.644 10.1001/jama.281.7.644 10029127

[29] www.helsedirektoratet.no/veiledere/prioriteringsveiledere/fordoyelsessykdommer/tilstander-for-fordoyelsessykdommer/dyspepsi

[30] Ganguli I, Lupo C, Mainor AJ et al (2020) Association between specialist compensation and accountable care organization performance. Health Serv Res 55(5):722–728. 10.1111/1475-6773.13323. Epub 2020 Jul 27. PMID: 32715464; PMCID: PMC7518824 10.1111/1475-6773.13323 PMC7518824 32715464

